# Antibacterial and Antifungal Studies of Biosynthesized Silver Nanoparticles against Plant Parasitic Nematode *Meloidogyne incognita,* Plant Pathogens *Ralstonia solanacearum* and *Fusarium oxysporum*

**DOI:** 10.3390/molecules26092462

**Published:** 2021-04-23

**Authors:** Masudulla Khan, Azhar U. Khan, Nina Bogdanchikova, Diana Garibo

**Affiliations:** 1 School of Life and Basic Sciences, SIILAS, Jaipur National University, Jaipur 302017, India; azhar.u.kh@gmail.com; 2 Centro de Nanociencias y Nanotecnología, Universidad Nacional Autónoma de México, 22800 Ensenada, Baja California, Mexico; nina@cnyn.unam.mx; 3 CONACYT Research Fellow at Centro de Nanociencias y Nanotecnología, Universidad Nacional Autónoma de México, 22800 Ensenada, Baja California, Mexico

**Keywords:** silver nanoparticles, antibacterial studies, antifungal studies, strawberry waste, *Fusarium oxysporum*, *Ralstonia solanacearum*

## Abstract

The possibility of using silver nanoparticles (AgNPs) to enhance the plants growth, crop production, and control of plant diseases is currently being researched. One of the most effective approaches for the production of AgNPs is green synthesis. Herein, we report a green and phytogenic synthesis of AgNPs by using aqueous extract of strawberry waste (solid waste after fruit juice extraction) as a novel bioresource, which is a non-hazardous and inexpensive that can act as a reducing, capping, and stabilizing agent. Successful biosynthesis of AgNPs was monitored by UV-visible spectroscopy showing a surface plasmon resonance (SPR) peak at ~415 nm. The X-ray diffraction studies confirm the face-centered cubic crystalline AgNPs. The scanning electron microscopy (SEM) and transmission electron microscopy (TEM) techniques confirm the rectangular shape with an average size of ~55 nm. The antibacterial and antifungal efficacy and inhibitory impact of the biosynthesized AgNPs were tested against nematode, *Meloidogyne incognita*, plant pathogenic bacterium, *Ralstonia solanacearum* and fungus, *Fusarium oxysporum*. These results confirm that biosynthesized AgNPs can significantly control these plant pathogens.

## 1. Introduction

Currently, around 1300 nanomaterials, with widespread potential applications, are currently available [1]. Silver nanoparticles (AgNPs) are a promising candidate in nanomedicine [2,3] and other AgNPs combinations, like those with biomaterials [4] to reduce microbial contamination. Globally in 2010, it is estimated that 260,000–309,000 metric tons of nanoparticles were produced [5], and worldwide consumption of nanomaterials was approximately 225,060–585,000 metric tons in 2014 to 2019 [6]. In nanomaterial science, green synthesis of nanomaterials has gained extensive attention as a sustainable, reliable, and eco-friendly protocol [7,8]. Plant extract can be used for synthesis of AgNPs. Plant extract of *R.* officinalis, U. dioica, and *V. vitis-idaea* were used for AgNPs synthesis [9]. Extract of alga *Parachlorella kessleri* can be used for AgNPs synthesis and they have antimicrobial potential [10,11]. Green synthesis of nanomaterials is an important alternative tool to reduce the destructive effects associated with the traditional methods of synthesis for nanoparticles commonly utilized in laboratory and industry settings [12,13,14,15]. The possibility of using nanomaterials to enhance the plants growth, crop production, and control of plant diseases is currently being researched all over the world with renewed vigor [16]. In recent years, nanotechnology has emerged as one of the most important fields of modern sciences [17,18]. Taniguchi in 1974 used the term nanotechnology to define the knowledge that deals with synthesis and application of nanosized particles (1–100 nm) of any material [19]. Nanotechnology has been increasingly applied to the development of antimicrobial materials for the management of pathogenic bacteria affecting agricultural crops, human beings, and animals. Significant development in nanomaterials synthesis, such as polymeric, carbon-based, and metallic NPs has attracted researcher attention for applications in managing plant diseases caused by pathogens [20]. AgNPs show the antimicrobial activity and gain lot of attention because of their unique antimicrobial, physiochemical, catalytic, and optical properties, as well as nanopharmaceuticals [21,22,23,24]. AgNPs show the inhibitory activity against many plant pathogenic fungi [25]. The plants, or plant extracts, which act as reducing and capping agents for NPs synthesis, are more advantageous, simple, and less prodigal over other biological primary sources [26]. Plant-mediated synthesis of NPs is preferred because it is a cost-effective, practical, environmentally friendly, and safe one-step method [27,28,29].

As agriculture is currently facing major challenges, due to climate change and high human population growth, it is difficult to meet the food demand so nanomaterials appear to be a useful tool for improving production and yields. Nanomaterials can be used to improve agricultural production by increasing the plant growth parameters.

Every year high crop loss occurs due to plant diseases caused by various pests and pathogens. *R. *solanacearum** is a Gram-negative bacterium which causes wilt to more than 150 plant species of 33 different plant families. It is responsible for great economic losses Worldwide [30]. *F. oxysporum* fungus represents a complex species that includes many important plant and human pathogens [31]. Disease caused by *Fusarium* spp., especially by *Fusarium* wilt (FW) in many crop plants, is one of the most intensively studied plant diseases. Root knot nematode (*M. incognita*) also causes root knot disease in many important crops. Currently, various plant disease management approaches mainly relies on toxic fungicides and pesticides that are potentially noxious to environment and human health. The latest approach in management of plant diseases is the use of NPs, which may be very fruitful. NPss can be used as alternatives to pesticides, as nanopesticides [32].

In order to fully utilize the advantage of nanotechnology in plant disease protection and management, it becomes essential to analyze the effect of nanosized particles on microbes and also their application in synthesizing fungicides and pesticides. By keeping it in our mind, in this article we report a green, handy, and environmentally friendly approach for the biosynthesis of AgNPs, using aqueous extract of strawberry waste (solid waste after fruit juice extraction) as a novel bioresource, which is non-hazardous and inexpensive. Additionally, we analyzed the impact of biosynthesized AgNPs on root knot nematode *M. incognita*, bacterium *R. solanacearum,* and fungus *F. oxysporum*.

## 2. Results and Discussion

### 2.1. XRD Analysis of the Synthesized AgNPs

**Figure 1:**
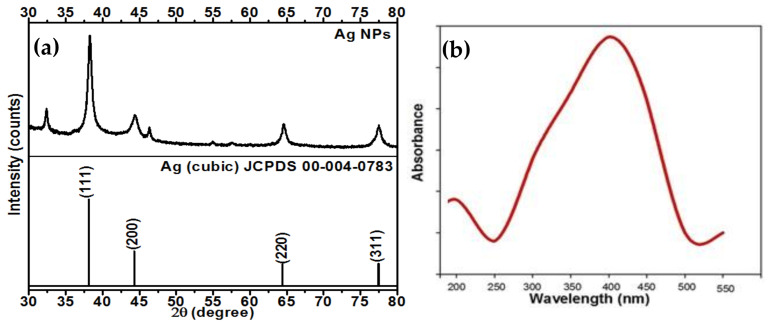
(**a**) XRD pattern and (**b**) UV-vis absorption spectrum of the biosynthesized AgNPs using aqueous extract of strawberry waste.

An X-ray diffraction pattern of the synthesized AgNPs was recorded at 10–80° 2θ range, as shown in Figure 1a. It was carried out to confirm the formation, structure, and crystalline nature of the synthesized AgNPs. The XRD pattern shows the prominent diffraction peaks at 38.4°, 46.3°, 64.7°, and 78.2°, which correspond to the (111), (200), (220), and (311) planes of metallic silver, respectively [21,23]. These peaks are well attributed to the standard JCPDS data of the silver with face-centered cubic (fcc) crystal lattice structure (JCPDS No. 00-004-0783) [21,23]. This confirms the polycrystalline nature of the synthesized AgNPs. The average crystallite size was calculated using Scherrer’s formula and found to be ~55 nm [21,23].

### 2.2. UV-Vis Spectroscopic Analysis of the Synthesized AgNPs

UV-Vis absorption spectroscopic analysis was performed to confirm the biosynthesis of AgNPs. It was observed that a colorless solution of AgNO_3_ starts changing to wine red color after 30 min, due to the formation of AgNPs. The UV-vis spectrum of the synthesized AgNPs is shown in Figure 1b. It showed an absorption band at ~415 nm, which is a characteristic band of surface plasma resonance (SPR) of AgNPs [21,23].

### 2.3. SEM of the Biosynthesized AgNPs

**Figure 2:**
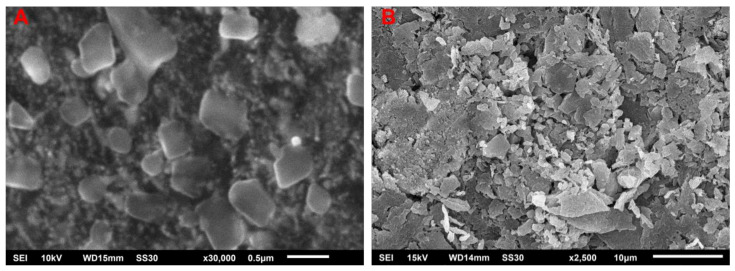
(**A**,**B**) SEM image of the biosynthesized AgNPs at different magnification showing rectangular shapes.

SEM analysis at different magnification of the biosynthesized AgNPs was carried out to investigate their morphology and size. SEM (Figure 2A,B) showed biosynthesized AgNPs with an almost rectangular shape. AgNPs aliquot prepared the coverslip and fixed the coverslip on an aluminum stub observed by SEM, as previously described by [33]. A sample of bacteria for SEM analysis was prepared by centrifugation of a broth culture of bacteria. Pellet was isolated and washed with phosphate buffer. 0.25% gluteraldehyde was added and left for some time. Samples were dehydrated by an ethanol series. Samples were added on SEM stub using two-sided tape.

### 2.4. TEM and SAED Studies of the Biosynthesized AgNPs

**Figure 3:**
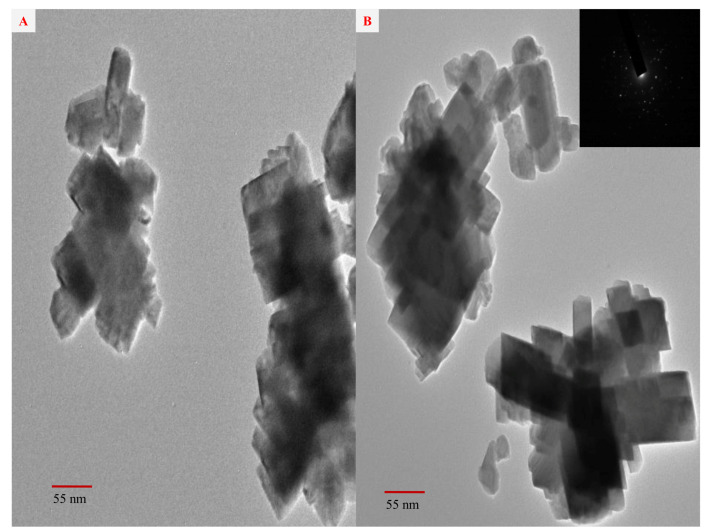
(**A**,**B**) TEM images (SAED as inset) of the biosynthesized AgNPs showing rectangular shapes.

TEM analysis was performed to study and confirm the morphology and size of the synthesized AgNPs. TEM analysis (Figure 3A,B) illustrates aggregates with an average size of 300 nm, consisting of AgNPs about 55 nm in size. The X-ray diffraction pattern of the synthesized AgNPs cannot provide information about particle size. AgNPs are in nanoscale, having the rectangular shape with average size of 55–70 nm. The selected area electron diffraction (SAED) pattern (Figure 3B) shows well-resolved lattice fringes, diffraction cycles, and concentric rings correspondingly indexed to the (111), (200), (220), and (311) planes of the “fcc” silver phase, which is the characteristic crystal planes of elemental Ag^0^ of highly crystalline nature of the biosynthesized AgNPs. The SAED patterns are in good agreement with the attributed XRD result, which also suggests the similar reflections of the synthesized AgNPs.

### 2.5. FTIR Analysis of the Biosynthesized AgNPs

**Figure 4:**
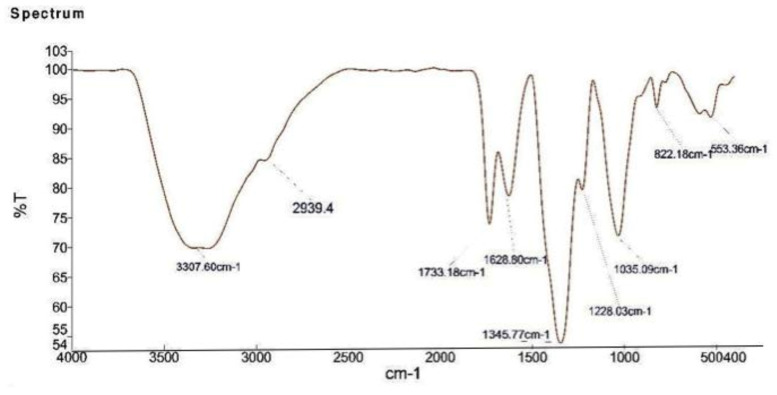
FTIR spectrum of the biosynthesized AgNPs.

FTIR spectroscopic analysis was performed to ascertain the involvement of possible phytochemicals and bio molecules used in the synthesis. Figure 4 shows the FTIR spectrum of obtained samples manifesting prominent transmittance peaks located at 3307 cm^−1^ (NH), 1733 cm^−1^ carbonyl group (C=O), and 1628 cm^−1^ (stretching -C=C-) attributed to aromatic ring stretching vibrations, respectively. These peaks suggested the presence of flavonoids and other phenolics in the extract [23]. The presence of flavonoids and other phenolics in the aqueous extract of strawberry waste could be responsible for the bio reduction of Ag^+^ ions and formation of AgNPs. The FTIR spectra (Figure 4) of AgNPs show stretching frequency bands at 3307 cm^−1^, 1628 cm^−1^, 1228 cm^−1^, 1035 cm^−1^, and 553 cm^−1^. Consequently, the occurrence of these peaks in the FTIR spectrum of AgNPs evidently indicates the dual role of the strawberry waste (peel) extract, both as a green reducing agent and also as a stabilizing agent.

### 2.6. In Vitro Antifungal and Antibacterial Activity of AgNPs

**Figure 5:**
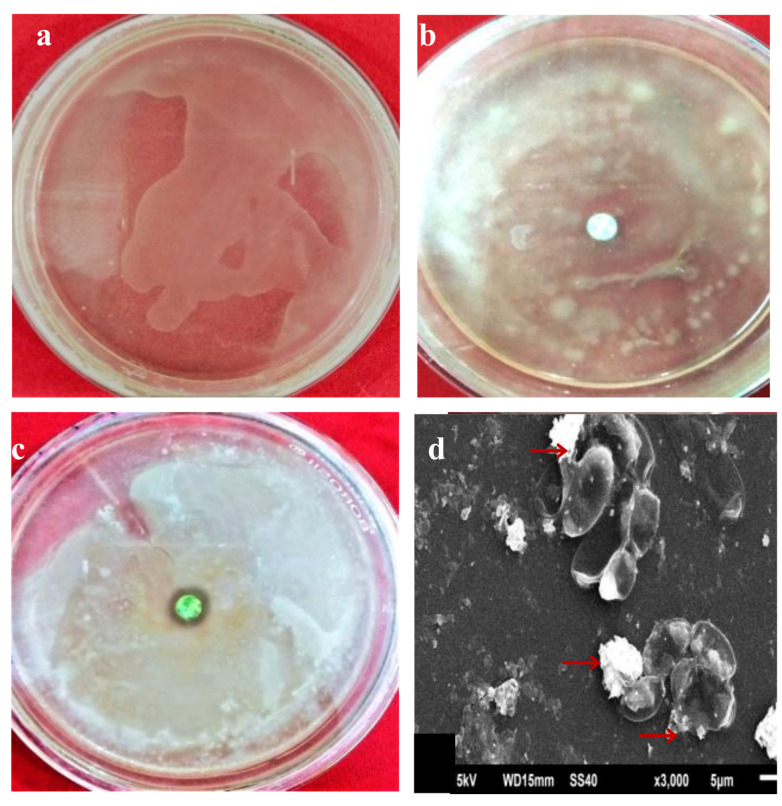
Antibacterial activity, (**a**) bacteria on nutrient agar media, (**b**) no inhibition zone (media without AgNPs), (**c**) Inhibition zone present in AgNPs treated media, and (**d**) SEM image showing disrupted and dead bacteria cells.

Until now, only a few articles including (at the same publication) results of antinematode, antifungal, and antibacterial studies against plant pathogens using AgNPs have been reported [23,34,35,36,37,38,39,40,41]. Therefore, in this work, we screened the biosynthesized AgNPs against the plant pathogenic bacterium, *R. solanacearum,* and fungus, *F. oxysporum*. As a result, we found a strong inhibition zone around the paper disc dipped in 100 µg/mL AgNPs, placed in nutrient agar medium inoculated with *R. solanacearum* and the inhibition zone is absent around the control disc (without biosynthesized AgNPs) (Figure 5b,c). The presence of the inhibition zone, induced (Figure 5c) by biosynthesized AgNPs, proves their antibacterial activity. The SEM analysis of the treated *R. solanacearum* was used to observe the morphological changes on the surface of the bacteria treated with AgNPs. Figure 5d shows the bacterial cell disruption after treating with the synthesized AgNPs. Khan et al. reported the similar antibacterial activities of the AgNPs [23]. This study confirms that AgNPs exhibited antimicrobial activities by damaging the cell membranes.

**Figure 6:**
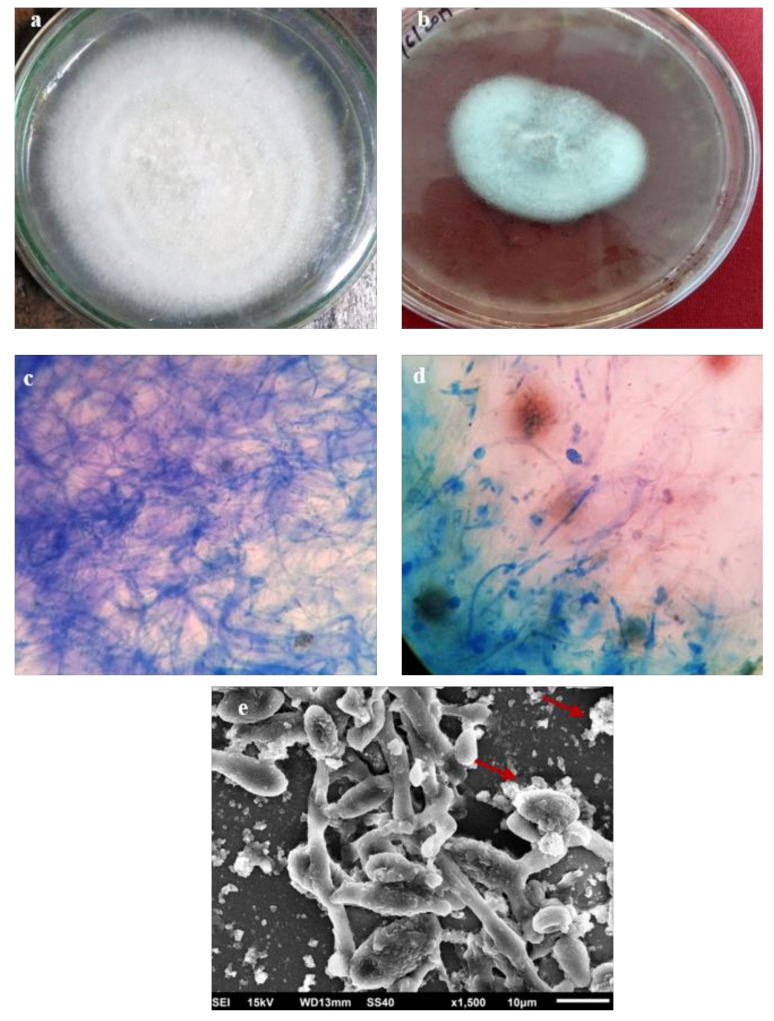
Antifungal activity, (**a**) *F. oxysporum* on PDA media without AgNPs, (**b**) F. oxysporum on PDA media with AgNPs, (**c**) fungal mycelium and, (**d**) fungal mycelium treated with AgNPs, and (**e**) SEM image showing ruptured fungal mycelium by AgNPs.

Similarly, biosynthesized AgNPs were tested against the plant pathogenic fungi, *F. oxysporum* (Figure 6a–e). In vitro studies show that AgNPs inhibited the mycelial growth of the fungus, *F. oxysporum*. The growth of *F. oxysporum* was considerably reduced to 40–50%.

Figure 6c shows fungal mycelium in potato dextrose medium (control without AgNPs) and Figure 6d shows fungal mycelium treated with 100 µg/mL AgNPs. Similar work has been reported by Khan et al., as well as Ghazy et al., in which antifungal activities of the AgNPs were observed [23,35]. The SEM analysis (Figure 6e) of the treated *F. oxysporum* was used to observe the morphological changes on the surface of the mycelium treated with AgNPs. Disruption of fungal mycelium has been observed at several places. Khan et al. has reported antifungal effects of AgNPs against plant pathogenic fungi and has observed a similar fatal effect on fungal mycelium [23]. This confirms that the biosynthesized AgNPs can be used as an antifungal agent to minimize their negative impacts on crop plants.

The results of the present study showed that AgNPs have antibacterial and antifungal activity in vitro against *R. solanacearum* and *F. oxysporum,* respectively. This finding is consistent with previous studies, which showed that AgNPs are effective for killing phytopathogens (including fungi, Gram-positive bacteria, and Gram-negative bacteria) (Table 1) [23,35,36,37,38,39,40,41]. Not all of them have carried out a basic characterization of the biogenic AgNPs obtained, which makes it difficult to explain the mechanism by which AgNPs interact with these microorganisms. Mishra et al. synthesized AgNPs and found that AgNPs can manage spot blotch disease in wheat [38]. Gupta and Chauhan reported fungicidal properties of AgNPs against *Alternaria brassicicola* fungus that causes Black Spot of cauliflower and radish [42]. Khan et al. synthesized AgNPs from corn and reported antibacterial and antifungal activity [35]. Ocsoy et al. has shown the effect of DNA-Directed AgNPs on Graphene Oxide (GO) as an antibacterial against bacteria, *Xanthomonas perforans.* Bacterial spot, caused by *X. perforans,* is a major disease of tomatoes, leading to a reduction in production by 10–50%. DNA-directed AgNPs grown on GO effectively decrease *X. perforans* cell viability in culture and on plants. At 16 ppm, these composites show excellent antibacterial capability. Application of these composites at 100 ppm on tomato transplants in a greenhouse experiment significantly reduced the severity of bacterial spot disease, compared to untreated plants [20].

AgNPs release silver ions inside the bacterial cells and enhance their bactericidal activity [43]. The impact of AgNPs on the antibacterial and antifungal activity depends on the size and shape of the synthesized NPs. From this study, it was observed that the biosynthesized AgNPs exhibit antibacterial and antifungal activities by damaging the cell membranes.

### 2.7. Effect of AgNPs on Nematode M. Incognita

In vitro studies showed that synthesized AgNPs could inhibit the hatching of *M. incognita* from eggs. AgNPs alone reduces the hatching (Table 2). Hatching of nematodes occurs highest in double distilled water at 24 and 48 h. Synthesized AgNPs show a toxic effect on nematodes and cause the death of hatched nematodes after 48 h of hatching. As the exposure time increases from 24 h to 48 h, activity of nematodes decreases. Lowest death of nematodes reported in double distilled water after 48 h. Previous studies also reported the toxic effect of biogenic AgNPs on nematodes [41]. Other works also reported toxic effect of the chemical synthetized AgNPs on *M. incognita* [44,45].

## 3. Experimental

### 3.1. Reagents

AgNO_3_ (GR93200), potato dextrose agar (PDA) and all other chemicals, reagents, and solvents used in this work were purchased from Merck India Ltd., Mumbai, India, and were used without any purification. Fungus, *F. oxysporum* (MTCC 1755) and bacteria *R. solanacearum* (BI0001) were used for screening the antifungal and antibacterial activity, respectively. They cultured on selected media for use.

### 3.2. Instruments

To confirm the synthesis of AgNPs, X-ray diffraction (XRD, PAN analytical, X’pert PRO-MPD, Almelo, Netherlands) was performed using CuKα radiation (λ = 0.15405 nm). UV-Vis spectral analysis was performed using Shimadzu spectrophotometer (UV-vis 1800, Kyoto, Japan). The morphology and size of the biosynthesized AgNPs were analyzed by scanning electron microscopy (SEM, NOVA nano FE-SEM 450 FEI, Hillsboro OR, USA) and Transmission electron microscopy (TEM, TECNAI-G-20, Hillsboro, OR, USA). Fourier transform infrared (FTIR) spectra of the AgNPs were obtained in the range of 4000–400 cm^−1^ with an FTIR spectrophotometer (Perkin Elmer Spectrum 2000 FTIR, Waltham, MA, USA) using KBr pellets.

### 3.3. Preparation of Aqueous Extract of Strawberry Waste

Strawberry waste (solid waste left after fruit juice extraction) was used to synthesize AgNPs on the basis of cost effectiveness, ease of availability, and environment friendliness. The waste material was cleaned with distilled water to remove contaminated contents and air-dried in sunlight. Material was crushed into a fine powder by a grinder and 20 mg fine powder was kept in a flask containing 200 mL of double distilled water and refluxed for 30 min. The extract was cooled down at room temperature and filtered with Whatman filter paper no.1 for further process.

### 3.4. Biosynthesis of AgNPs Using Aqueous Extract of Strawberry Waste

An aqueous solution of 1 mM AgNO_3_ of 100 mL was prepared in an Erlenmeyer flask. A 10 mL of strawberry extract was added to 90 mL of this AgNO_3_ aqueous solution and left at room temperature with continuous stirring until color changes were apparent. During this, reduction of Ag^+^ to Ag^0^ was observed by the color change of solution from colorless to wine red. AgNPs formation was further confirmed by UV–visible spectroscopy.

### 3.5. Bacterial Inoculum

Bacterium, *R. solanacearum* was cultured on nutrient agar medium. Nutrient agar plates were streaked separately with a pure colony of *R. solanacearum* and incubated at 30 °C for 24 h [23,46]. Antibacterial activity of biosynthesized AgNPs was screened against *R. solanacearum*. A small paper disc was dipped in a 100 µg/mL AgNPs solution and placed in media containing *R. solanacearum* and compared with control (paper disc without NPs). These plates were incubated at 35 °C for ~24 h, after which, diameters of the inhibition zones were measured. Presence of inhibition zone is a sign of antibacterial activity. All the tests were run in triplicate.

### 3.6. Fungus Inoculum

Fungus, *F. oxysporum*, was grown in Petri dishes containing potato dextrose agar (PDA) medium at 25 °C for 15 days. For obtaining sufficient inoculum, *F. oxysporum* was cultured on Richard’s liquid medium [47]. Antifungal activity was evaluated by poisoned food technique [48,49] by using biosynthesized AgNPs. The treated plates were compared with the control to calculate the percent inhibition of mycelia growth by using the formula given in [50]. Experiments were carried out in triplicate.

Fungal mycelium inhibition (FMI):

(1) $$\mathrm{FMI}\;(\%)=\frac{R-r}{R}\cdot 100$$

where “*R* = Mycelia growth in control” and “*r* = mycelia growth after treatment with AgNPs”.

### 3.7. Root Knot Nematodes

**Figure 7:**
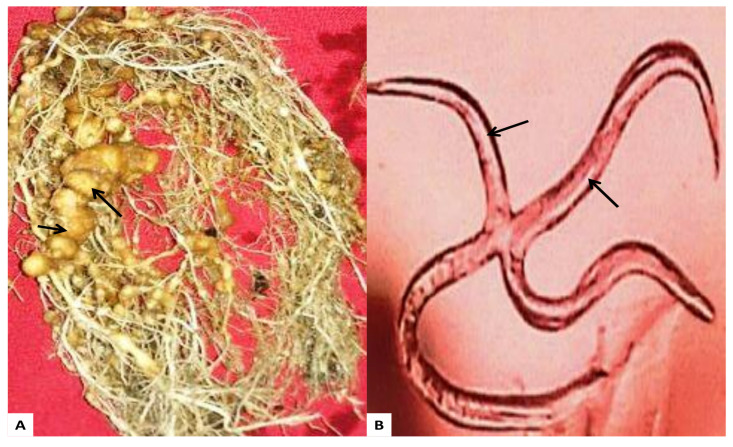
(**A**) Infected root caused by nematode *M. Incognita* (**B**) *M. incognita* juveniles.

Root-knot nematode, *M. incognita*, was isolated from infected roots and cultured (Figure 7). AgNPs were used to screening their effect on *M. incognita*. To determine the nematicidal activity of prepared AgNPs, 2.5 mL (100 µg/mL) suspension was dissolved in 7.5 mL distilled water in Petri plates. Five egg masses of nematodes were placed on each Petri plate. One Petri plate had five egg masses and 10 mL distilled water was used as a control (without AgNPs). 100 µg/mL solutions were prepared by dissolving 0.1 mL solutions of synthesized AgNPs in one liter of distilled water. All the tests were run in triplicate.

## 4. Conclusions

This study accomplishes the biosynthesis of AgNPs using aqueous extract of strawberry waste. The biosynthesized AgNPs showed antinematode, antibacterial, and antifungal response against *M. incognita*, *R. solanacearum,* and *F. oxysporum,* respectively, owing to considerable antibacterial and antifungal activities. The biosynthesized AgNPs showed acute, potent antifungal effects on fungi tested in vitro, probably through rupture or fissure of membrane integrity. Hence, we can accede that the biosynthesized AgNPs can be used against plant pathogens for retrieval of agricultural production, food safety, and a range of other applications.

## Figures and Tables

**Table 1:** Effect of AgNPs on phytopathogens.

Phytopathogens	Bioresource	Concentration of AgNPs	Stabilizer Type	Size of NPs	Zeta Potential, mV	FTIR Study	XRD Study	SPR Peak (nm)	AgNPs Effect on Phytopathogens	Reference
*Ralstonia solanacearum, Fusarium oxysporum* and *Meloidogyne incognita*	strawberry waste (solid waste after fruit juice extraction)	100 μg/mL	Secondary metabolites in extract	55–70 nm	n.d	Peaks located at 3307 (NH), 1733 carbonyl group (C=O), and 1628 (stretching -C=C-) aromatic ring stretching vibrations.	Peaks at 38.4°, 46.3°, 64.7°, and 78.2°, which correspond to the (111), (200), (220), and (311) planes of metallic silver.	~415	Inhibit growth of tested phytopa-thogens	Our work
*Alternaria alternate, Pseudomonas syringae*	Leaf *of the Trigonella foenum-graecum*	100 μg/mL	Secondary metabolites in extract	20–25 nm	n.d	Peaks 3247, 2919, 1587, 1376, and 1019 cm^−1^, which represents free OH, stretching -C=C- aromatic ring, and C-OH stretching vibrations, respectively	Peaks at 27.9°, 32.4°, 38.2°, and 46.3°, which correspond to the (111), (200), (220), and (311) planes, respectively.	~410	Inhibit growth of tested phytopa-thogens	[23]
*Pectobacterium carotovorum*	*Fusarium oxysporum*	n.d	Secondary metabolites in extract	16–27 nm	n.d	n.d	n.d	~430	Inhibit growth of tested phytopa-thogens	[35]
*Phomopsis vexans, Ralstonia solanacearum*	Corn seeds	100 μg/mL	Secondary metabolites in extract	25 nm	n.d	Peaks at 3284 (OH), 1645, 1400, 1336–1145, which represents free OH in molecules and stretching -C=C- aromatic ring and C-OH stretching vibrations.	2θ values of 27.91°, 32.19°, and 46.64° sets of lattice planes.	423, 437, 464	Inhibit growth of tested phytopa-thogens	[36]
*Fusarium oxysporum*	Leaf extract of *Melia azedarach*	n.d	Secondary metabolites in extract	12–46 nm	−22.3	Peaks at 3258.25 and 1634.31 cm^−1^ represents vibrations of hydroxyl (–OH) group and alkene (C=C) with aromatic ring, respectively	Peaks at 38.12°, 44.23°, 64.51°, and 77.69° that can be assigned to the plane of (111), (200), (220), and (311), respectively	434	Inhibit growth of tested phytopa-thogens	[37]
*Bipolaris sorokiniana*	*Serratia* sp.	n.d	Secondary metabolites in extract	10–20 nm	n.d	Peaks at 3436.52 and 2942.73 cm^−1^ were assigned to the stretching vibrations of primary and secondary amines, respectively.	Bragg reflections were obtained at 2θ = 38.4°, 44.5°, 64.6°, and 76.9°, which correspond to the crystal lattice planes (111), (200), (220), and (311) of face centered cubic (*fcc*) structures of silver (JCPDS files No. 03-0921), respectively	410	Inhibit growth of tested phytopa-thogens	[38]
*Bacillus megaterium, Pseudomonas syringae, Burkholderia glumae, Xanthomonas oryzae, and Bacillus thuringiensis*	Pine cone	108 μg/mL	Secondary metabolites in extract	5–50 nm	30	Peaks at 3449 (vibrations of the O–H groups), 2922 (asymmetric and symmetric C–H stretching), 1718 (carbonyl stretching), 1615, and 1509 (asymmetrical stretching of the carboxylate group), 1369 (symmetrical stretch of the carboxylate group), 1263 (acetyl group), 1160, and 1057 (C–O stretching vibration of ether and alcohol groups), and 3449–3406 cm^−1^ (binding of silver ions with hydroxyl groups).	Peaks at 38.6°, 44.2°, 46.2°, 65.2°, 68.1°, 78.2°, and 85.2°. The peak at 85.2° was unidentified and indexed to the 111, 200, 220, and 311 planes of the cubic face-centered silver	414	Inhibit growth of tested phytopa-thogens	[39]
*Botrytis cinerea, Alternaria alternata, Curvularia lunata, Rhizoctonia solani, Macrophomina phaseolina, Sclerotinia sclerotiorum*	*Acalypha indicaleaf*	95 μg/mL	Secondary metabolites in extract	10–50 nm	n.d	n.d	Peaks at 38.1°, 44.1°, and 64.1°, which indexed the planes 111, 200, and 220 of the cubic face-centered silver	n.d	Inhibit growth of tested phytopa-thogens	[40]
*Meloidogyne incognita*	*Urtica uren leaf*	n.d	n.d	Ethyl acetate extract: 60–112 nm) Ethanol extract: 80–111 nm	n.d	n.d	n.d	n.d	Inhibit growth of tested phytopa-thogens	[41]

n.d = no data.

**Table 2:** Effect of AgNPs on nematode, *M. incognita*.

Treatments	No. of M. *incognita* Hatched after 24 h	No. of M. *incognita* Hatched after 48 h	No. of M. *incognita* Dead after 48 h
Distilled water	38	81	04
AgNPs + Distilled water	24	47	09
